# Bacterial Quorum Sensing Allows Graded and Bimodal Cellular Responses to Variations in Population Density

**DOI:** 10.1128/mbio.00745-22

**Published:** 2022-05-18

**Authors:** Jennifer B. Rattray, Stephen A. Thomas, Yifei Wang, Evgeniya Molotkova, James Gurney, John J. Varga, Sam P. Brown

**Affiliations:** a School of Biological Sciences, Georgia Institute of Technology, Atlanta, Georgia, USA; b Center for Microbial Dynamics and Infection, Georgia Institute of Technology, Atlanta, Georgia, USA; c Graduate Program in Quantitative Biosciences (QBioS), Georgia Institute of Technology, Atlanta, Georgia, USA; d The Institute for Data Engineering and Science (IDEaS), Georgia Institute of Technology, Atlanta, Georgia, USA; Emory University

**Keywords:** bacterial communication, quorum sensing, reaction norm, sociomicrobiology

## Abstract

Quorum sensing (QS) is a mechanism of cell-cell communication that connects gene expression to environmental conditions (e.g., cell density) in many bacterial species, mediated by diffusible signal molecules. Current functional studies focus on qualitatively distinct QS ON/OFF states. In the context of density sensing, this view led to the adoption of a “quorum” analogy in which populations sense when they are above a sufficient density (i.e., “quorate”) to efficiently turn on cooperative behaviors. This framework overlooks the potential for intermediate, graded responses to shifts in the environment. In this study, we tracked QS-regulated protease (*lasB*) expression and showed that Pseudomonas aeruginosa can deliver a graded behavioral response to fine-scale variation in population density, on both the population and single-cell scales. On the population scale, we saw a graded response to variation in population density (controlled by culture carrying capacity). On the single-cell scale, we saw significant bimodality at higher densities, with separate OFF and ON subpopulations that responded differentially to changes in density: a static OFF population of cells and increasing intensity of expression among the ON population of cells. Together, these results indicate that QS can tune gene expression to graded environmental change, with no critical cell mass or “quorum” at which behavioral responses are activated on either the individual-cell or population scale. In an infection context, our results indicate there is not a hard threshold separating a quorate “attack” mode from a subquorate “stealth” mode.

## INTRODUCTION

Many species of bacteria are capable of a form of cell-cell communication via diffusible signal molecules, generally referred to as quorum sensing (QS). The study of QS has largely focused on the intracellular gene regulatory scale, leading to a detailed understanding of the regulatory mechanisms shaping the production of and response to signal molecules in model organisms such as Vibrio cholerae, Bacillus cereus, and Pseudomonas aeruginosa (1–3). We now understand that QS is mediated by multiple diffusible signals that together control a diverse array of responses, including swarming, luminescence, competence, and the production of diverse secreted factors (4, 5).

While the molecular mechanisms of QS have been described for model organisms in remarkable detail, the functional and evolutionary context of QS continues to be disputed. In other words, while we now have a better understanding of how QS works, we still have limited understanding of why bacteria use this system to control behavior. What are the functions of QS? How do these QS functions help bacteria to survive and grow? The standard answer is that bacteria use QS to sense when they are at sufficient density (“quorate”) to efficiently turn on cooperative behaviors such as secretion of toxins and enzymes in order to collectively modify their environment (6–8). Other researchers have argued that QS is a device to sense the physical environment, where individual cells produce and monitor signal levels in order to infer their local physical constraints (am I in an open or enclosed space?) (9). More recently, integration of molecular and evolutionary approaches has increased the menu of potential functions to include sensing multiple aspects of both the social and physical environments (6, 10–12) and coordinating complex social strategies that limit the profitability of noncooperating “cheat” strains (13–22).

**FIG 1:**
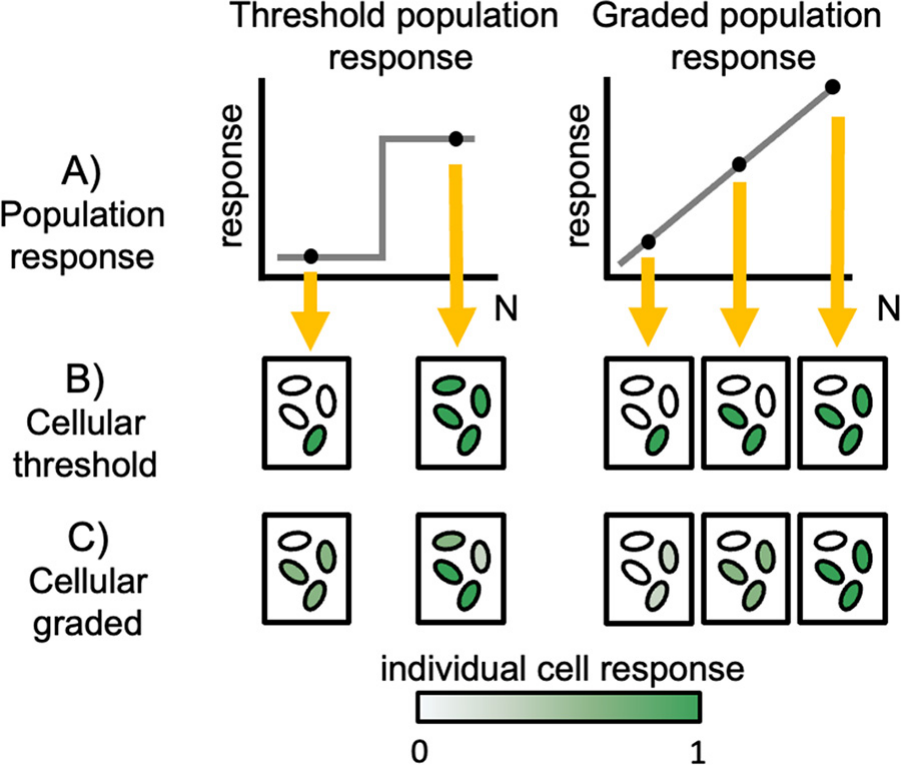
Schematic of potential population and single cell responses to variation in cell density. (A) Population response (*y* axis) across discrete carrying capacity environments (N, *x* axis), given a threshold (left) or graded response (right). In panels B and C, we outline alternative cell-scale responses (intensity of green cells) that are consistent with discrete population scale behaviors (yellow arrows). (B) Threshold (ON/OFF) cellular responses can produce a threshold or graded responses on population scale. (C) Individual responses can be threshold or graded, which can produce threshold or graded responses on a population scale.

A critical step in assessing the various adaptive hypotheses is establishing the functional capacities and limits of QS. The studies outlined above largely focus on a dichotomy of QS ON/OFF (or, quorate/subquorate) states, overlooking the potential for intermediate, graded responses (Fig. 1A). The threshold quorate/subquorate concept has support from mathematical models of QS signal dynamics, which highlight how positive feedback on the signaling molecule can produce a sharp threshold response to changes in environmental parameters such as density or diffusion (23, 24). However, the same mathematical models indicate that graded responses are also possible, dependent on the model parameterization (23, 24). More generally, Fig. 1A highlights that the phenotypic response of QS bacteria to differing environmental conditions can be viewed as a “reaction norm” (25–28) that can in principle take differing shapes. Reaction norms describe phenotypic responses of a single genotype (*y* axis in Fig. 1A) to various environmental inputs (*x* axis in Fig. 1A). Incorporating a reaction norm framework provides a menu of quantitative metrics to define QS responses to environmental variation (e.g., slope, intercept, and variances). With this reaction norm framework, it is important to emphasize that in our study the *x* axis is not time but instead captures a gradient of environmental conditions. Whether responses are graded or thresholded during the growth toward high density is a separate line of inquiry (29). Describing the reaction norms of QS cells and populations to contrasting environments is an important step toward understanding the capacities of QS systems to differentially respond to novel environments.

Whether the population scale reaction norm to environmental variation is threshold-like or graded (Fig. 1A), a separate issue is how collective population-level responses are constructed out of individual cellular contributions (Fig. 1B and C). Studies of QS on a single-cell scale have revealed substantial heterogeneity in responses to QS signals (11, 30–41), highlighting that cell-cell communication does not necessarily result in tight synchronization of individual-cell activities (Fig. 1B and C). In some systems, heterogeneity can be quenched by the addition of extra signal (35, 41), implying a lack of receptor saturation. However, this is not a universal result (40), indicating that other molecular processes can drive cellular variation in response. Regardless of the molecular details, we currently lack a behavioral understanding of how individual cellular responses vary with changes in the environment.

In the current study, we addressed the canonical “density sensing” function of QS, using the environmental generalist and opportunistic pathogen Pseudomonas aeruginosa and an unprecedented scale of environmental resolution (13 discrete limiting carbon levels conducted in triplicate, generating 39 density environments). QS in Pseudomonas aeruginosa is heavily studied in a high-density (ON/OFF) context, revealing a complex mechanism of multisignal control (42–46). Our first challenge was to map the population scale resolving power of QS to quantitatively discriminate graded differences in population density (Fig. 1A). Does P. aeruginosa respond in a purely threshold manner, collapsing quantitative differences in population density into a simple low/high qualitative output, or can QS allow P. aeruginosa to deliver a graded response to distinct environmental densities? Our second challenge was to understand how collective responses are partitioned across individual cells. Are changes in collective responses governed primarily by changes in the proportion of cells in an ON state (Fig. 1B), changes in the individual-cell intensity of response (Fig. 1C), or both?

## RESULTS

### Collective level of response to density is graded and linear.

**FIG 2:**
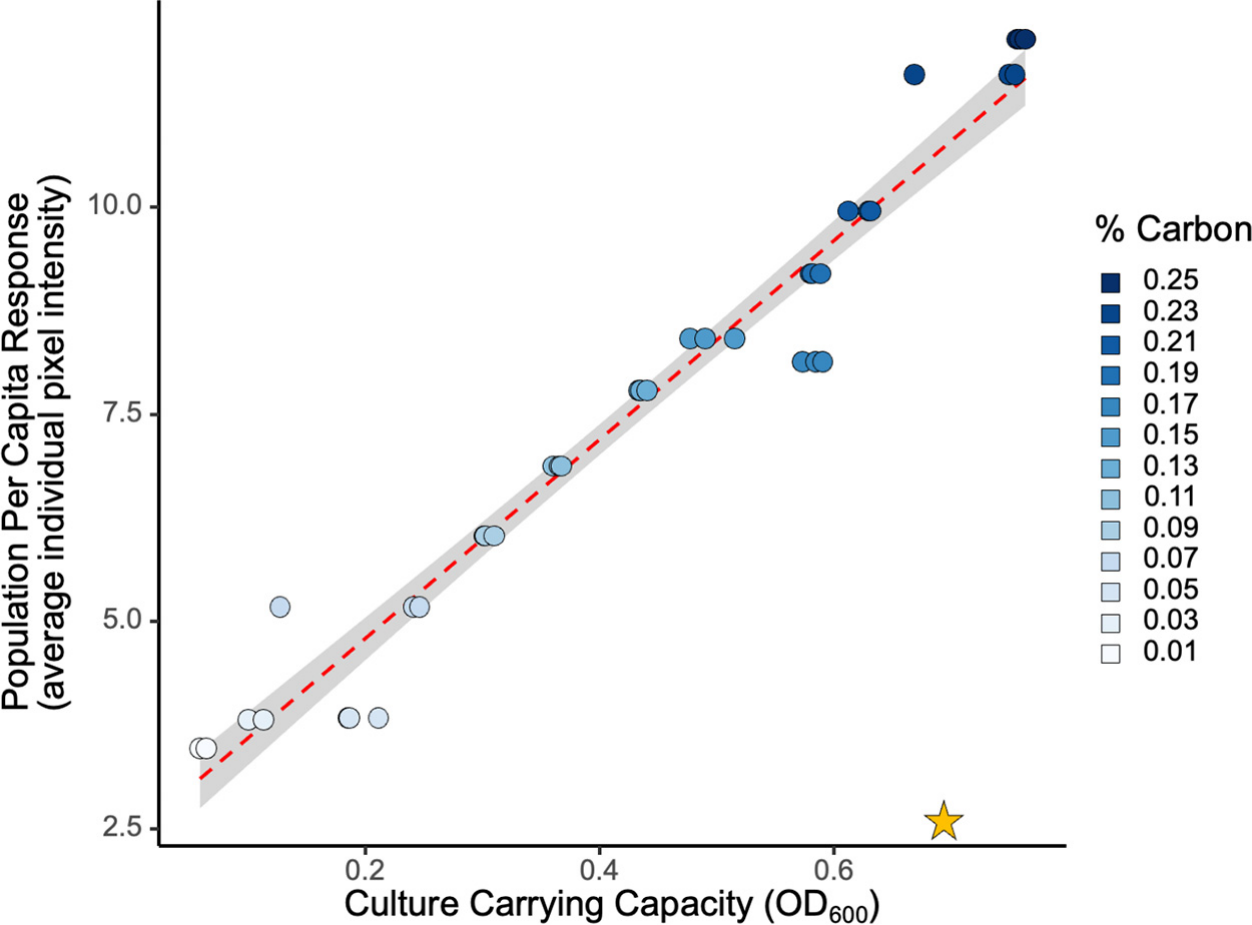
Population response to increasing cell density is linear and graded. Thirteen distinct culture carrying capacities were generated by manipulating the concentration of casein digest as the limiting resource (Fig. S1). Cells were grown to carrying capacity in triplicate and immediately assayed for quorum sensing (QS) response via fluorescence microscopy imaging. Response was determined by a fusion of the quorum sensing-controlled *lasB* promoter and an unstable green fluorescent protein [PAO1 pMHLAS containing *PlasB*::*gfp*(*ASV*)]. Individual cell pixel intensity is a measure of cellular QS response, and average pixel intensity was calculated across all cells in the population as a proxy for total population expression. Microscopy averages are congruent with population scale plate reader results (Fig. S2). A QS signal knockout (Δ*lasI* Δ*rhlI*; yellow star) shows background response with no signal in the environment. Average population investment in QS increased as culture density increased, with no observable density threshold (AIC linear, 89; AIC step function, 190).

Our first challenge was to map out the population scale reaction norm of the collective QS-controlled protease (encoded by *lasB*) response to variation in population densities. To provide a detailed picture of the QS response reaction norm to various densities, we grew a QS reporter strain [PAO1 pMHLAS containing the *PlasB*::*gfp*(*ASV*) reporter construct for QS-regulated protease expression (47)] under 13 conditions of carbon limitation in triplicate and measured average fluorescence output per cell as the populations reached carrying capacity (Fig. 2). Dead cells with compromised membranes were identified with a propidium iodide stain and excluded from analysis. The range of cell densities generated from this method was 1 × 10^8^ cells/mL to 2 × 10^9^ cells/mL. Figure 2 shows that QS response was linear with increasing culture density, providing intermediate levels of average per-capita response to intermediate densities. To confirm the lack of threshold behavior, we assessed alternate statistical models including threshold functions and found that a linear-fit model supports the data better than a step function fit (Akaike information criterion [AIC] linear, 89; AIC step function, 190; relative likelihood that the linear model is the better fit than step function, >10^9^; see reference 48), supporting a graded population response as outlined in Fig. 1. This agrees with literature reporting that QS induction at lower population densities is possible (6, 7, 11) but differs in that there is no observable population density at which populations “switch,” or reach quorum, into a responsive state.

10.1128/mbio.00745-22.1 FIG S1 Growth curves for PAO1 pMHLAS across different carbon-limiting environments. Discrete environmental densities can be generated by varying carbon availability, therefore manipulating the carrying capacity of the culture. Download FIG S1, DOCX file, 0.04 MB. Copyright © 2022 Rattray et al. 2022 Rattray et al. https://creativecommons.org/licenses/by/4.0/ This content is distributed under the terms of the Creative Commons Attribution 4.0 International license.

10.1128/mbio.00745-22.2 FIG S2 Microplate population per capita data is graded and linear. PAO1 pMHLAS was grown as mentioned in Materials and Methods in the main text, and fluorescence and OD_600_ were measured on a Cytation Sense plate reader. Microplate results agree with microscopy results that that population response to increasing cell density is linear and graded. An OD_600_ of 0.25 is 5.5 × 10^8^ cells/mL, an OD_600_ of 0.50 is 1.3 × 10^9^ cells/mL, and an OD_600_ of 0.75 is 2.05 × 10^9^ cells/mL. Download FIG S2, DOCX file, 0.1 MB. Copyright © 2022 Rattray et al. 2022 Rattray et al. https://creativecommons.org/licenses/by/4.0/ This content is distributed under the terms of the Creative Commons Attribution 4.0 International license.

### Individual response to density is bimodal at high densities.

**FIG 3:**
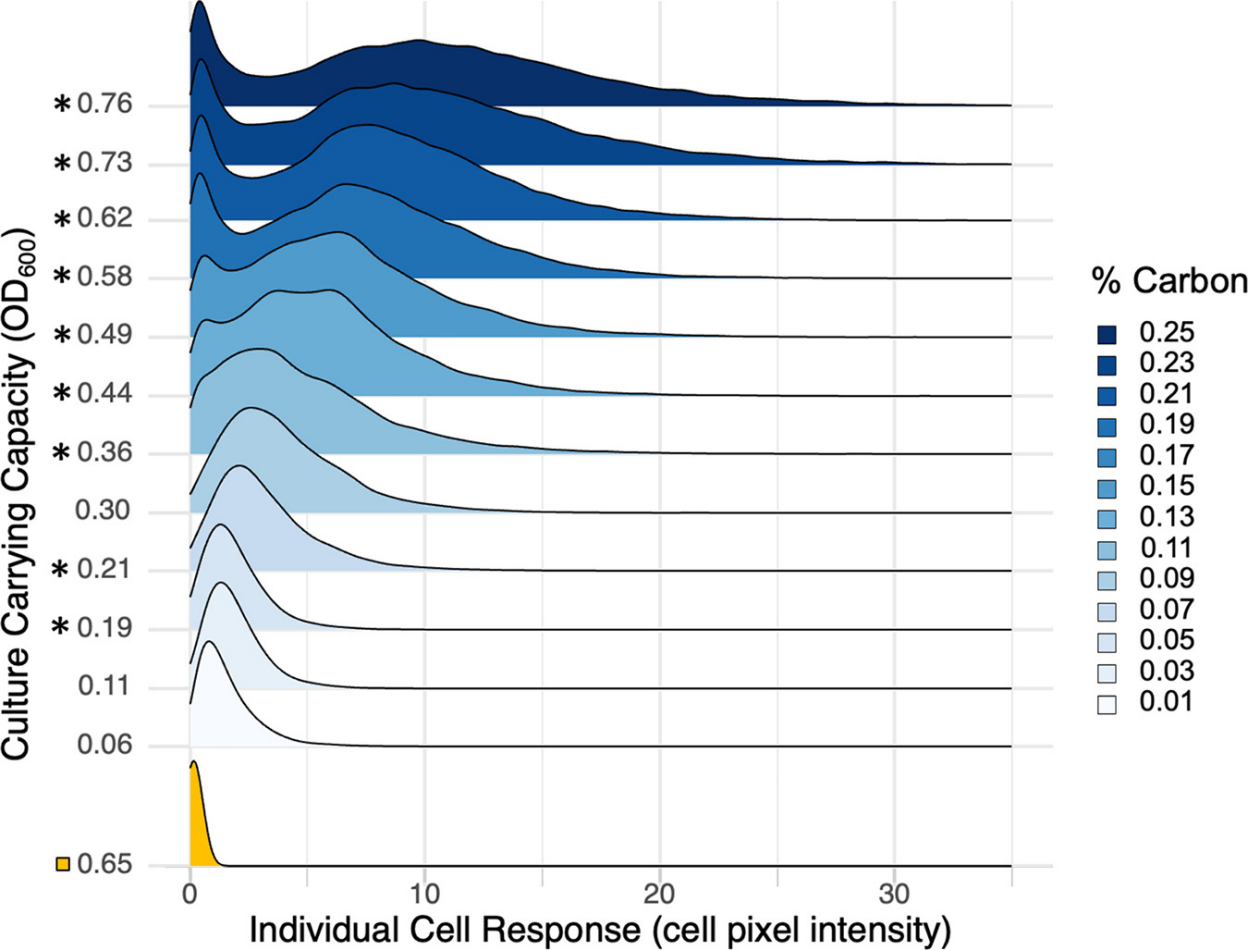
Individual response is heterogenous and bimodal at higher densities. A ridgeline density plot (bandwidth = 0.435) of single-cell *lasB* reporter response data shows the distribution of individual-cell QS expression across the population. For brevity and plotting purposes, carrying capacities were averaged across 3 replicates for each of the 13 carbon environments before plotting. A full plot of each independent replicate environment can be found in Fig. S3. Each line summarizes 18,000 to 30,000 individual-cell measurements, scaled to a unit height. Asterisks indicate significant bimodality (Hartigan’s dip test (71), Fig. S4). The QS signal knockout (Δ*lasI* Δ*rhlI*) is designated with a yellow box. A total of 345,000 individual-cell measurements were analyzed.

Figure 2 establishes that on a collective population scale, the response to environmental variation (in density) is smoothly graded. Next, we asked how this collective response is built from individual cell contributions. Is the graded increase due to more cells turning ON at higher densities (Fig. 1B), cells turning ON to a greater extent (Fig. 1C), or both? To address this question, we take the same data presented in Fig. 2 and now present the distribution of individual cellular responses rather than simply the mean response (Fig. 3).

10.1128/mbio.00745-22.3 FIG S3 Ridgeline density plot (bandwidth = 0.529) of single-cell *lasB* reporter response data showing the distribution of individual cell QS expression across the population. All 39 replicates are plotted separately. Asterisks indicate significant bimodality (Hartigan’s dip test [J. A. Hartigan and P. M. Hartigan, Ann Stat 13:70–84, 1985] [Fig. S4]). Download FIG S3, DOCX file, 0.2 MB. Copyright © 2022 Rattray et al. 2022 Rattray et al. https://creativecommons.org/licenses/by/4.0/ This content is distributed under the terms of the Creative Commons Attribution 4.0 International license.

**FIG 4:**
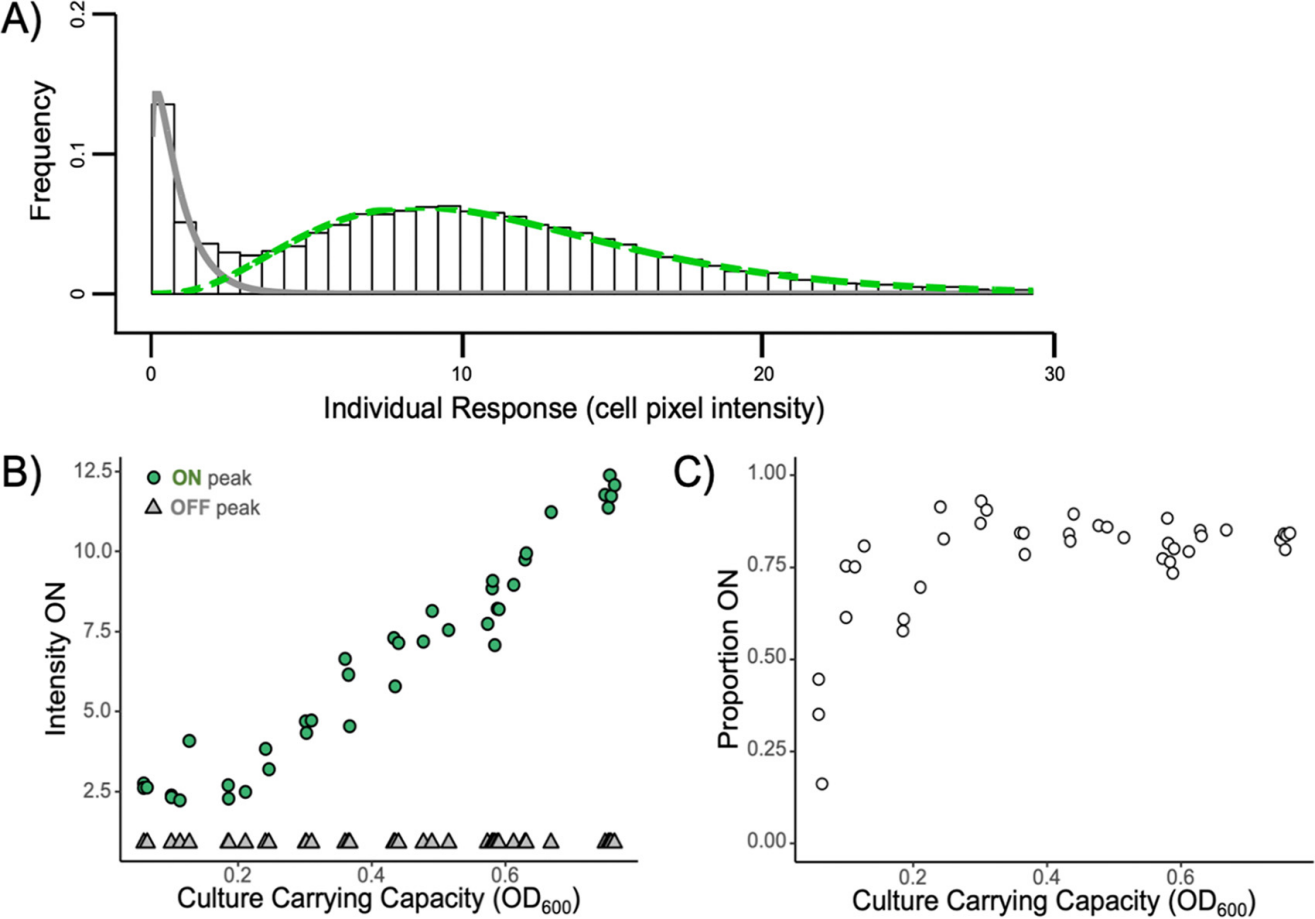
The proportion of cells responding and level of response varies with density. In light of the bimodal responses in Fig. 3, we course grained the single-cell *lasB* response data into discrete ON/OFF states. (A) Method summary. We quantified distinct ON/OFF states by fitting a two-component finite mixture model at each measured optical density, where the OFF state was fixed to the OFF state of the highest-density environment. The histogram shows the distribution of cellular expression levels at a single density treatment (OD_600_ of 0.76); the gray line is the fitted OFF state, and the green dashed line is the fitted ON state. (B) The mean intensity of the ON (green circles) and OFF (gray triangles) states was determined from the means of mixture model component fits (green and gray lines in panel A). The mean intensity of the ON state distribution increased as culture density increased, while the mean of the OFF state remained constant. (C) The proportion of cells ON in the population was determined from the relative mass of cells in the model component fits. The proportion ON increased with culture density but did not reach 100%.

10.1128/mbio.00745-22.4 FIG S4 Hartigan’s dip test. (A) Results of Hartigan’s dip test (Hartigan and Hartigan, Ann Stat 13:70–84, 1985) for assessing bimodality of the expression level distributions at each carbon level for data shown in Fig. 4. (B) Results of Hartigan’s dip test for assessing bimodality of the expression level distributions at each OD level for data shown in Fig. S3. Statistical significance in the form of *P* value is plotted in yellow on the left axis, and the dip metric is plotted in green on the right axis. The most conservative *P* value is shown. Download FIG S4, DOCX file, 0.1 MB. Copyright © 2022 Rattray et al. 2022 Rattray et al. https://creativecommons.org/licenses/by/4.0/ This content is distributed under the terms of the Creative Commons Attribution 4.0 International license.

As expected from prior studies in other QS systems (11, 30, 31, 34–36, 40, 41), plotting all individual responses within a population showed cell-to-cell variation in QS response within a single population despite isogenic and homogenous culture conditions (Fig. 3). In addition, at higher densities, we saw significant bimodality (defined by Hartigan’s dip test; see Fig. S4 in the supplemental material, with the population segregating into a responsive ON (“quorate”) state and an unresponsive OFF (“subquorate”) state).

In light of this bimodality, we fit a two-component finite mixture model to the data (Fig. 4A; see https://github.com/GaTechBrownLab/Rattray-2022 for extended analysis), which allows us to define the average intensity of the ON state (Fig. 4B) and the proportion of cells in the OFF or ON state (Fig. 4C).

Figure 4B illustrates a graded linear increase in the intensity of the ON state with increasing environmental density and a density-invariant OFF state. Figure 4C illustrates that the proportion of cells that are ON plateaus at around 85% at densities with consistent support for bimodality (above an optical density at 600 nm [OD_600_] of 0.36). At lower densities, the intensity of the ON state (Fig. 4B) declines to a point where the OFF and ON states are no longer significantly different and the dip test fails to reject unimodality (Fig. S4). These results depicted in Fig. 4B and C are consistent with a mix of the cellular threshold and cellular graded models outlined in Fig. 1, with both components contributing to the collective response in Fig. 2. In the supplemental material, we present alternate statistical analyses of these data and of other related data sets. Across other experiments, we found consistent support for the graded and bimodal response pattern on the single-cell scale across multiple assay time points (Fig. S5) and across two reporter strain constructs (Fig. S6) and support for the graded and linear response pattern on the population scale across fluorescent and *lux* reporters (Fig. S2 and S7). We found further support for the graded population response on the population scale across two additional QS-controlled genes (*pqsA* and *rhlI* [Fig. S7]).

10.1128/mbio.00745-22.5 FIG S5 Single-cell results are not sensitive to the exact time sampled. In order to test the generality of our results and how sensitive they are to the specific time sampled, we repeated our main experiment with PAO1 pMHLAS but took samples from five different time points instead of just entry into stationary phase. We observed the same bimodal response and shifts in proportion responding and level of response across all time points, concluding that our results are not sensitive to the exact measurement time. Download FIG S5, DOCX file, 0.2 MB. Copyright © 2022 Rattray et al. 2022 Rattray et al. https://creativecommons.org/licenses/by/4.0/ This content is distributed under the terms of the Creative Commons Attribution 4.0 International license.

10.1128/mbio.00745-22.6 FIG S6 Presence/absence of *Plac*::*lasR* and plasmid nature of the reporter do not impact graded QS response. The plasmid hosting our reporter, pMHLAS, also contains P*lac*::*lasR*. While the lac operon is typically repressed, some strains of Pseudomonas are capable of leaky, noninduced expression (J. Meisner and J. B. Goldberg, Appl Environ Microbiol 82:6715–6727, 2016, https://doi.org/10.1128/AEM.02041-16). To confirm our single-cell data, we repeated the experiment using a strain with the *PlasB*::*gfp*(*ASV*) reporter inserted with the mini-Tn*5* method. This strain does not contain *Plac*::*lasR* and expresses wild-type levels of *lasR*. We found the same bimodal response to density environments in both strains. This indicates that neither the presence of *Plac*::*lasR* nor the plasmid-based nature of our reporter was responsible for the graded QS response. This could mean either that there is not an appreciable amount of *lasR* being made from *Plac*::*lasR* or that the increase of *lasR* does not impact the observed response. These results confirm that the observed response across density is not sensitive to plasmid copy number or the presence of potentially leaky *Plac*::*lasR* with this specific reporter. Download FIG S6, DOCX file, 0.1 MB. Copyright © 2022 Rattray et al. 2022 Rattray et al. https://creativecommons.org/licenses/by/4.0/ This content is distributed under the terms of the Creative Commons Attribution 4.0 International license.

10.1128/mbio.00745-22.7 FIG S7 Population response to increasing cell density using chromosomally inserted lux based QS reporters (72–74). (A) Strain P NPAO1 mini-CTX *PlasB*::*lux*; (B) NPAO1 mini-CTX *PpqsA*::*lux*; (C) NPAO1 mini-CTX *PrhlI*::*lux*. Bacteria were grown following the methods outlined in Materials and Methods in the main text and monitored in a plate reader. Per-capita expression (relative light units [RLU]/OD_600_) was calculated by taking the maximum expression (RLU) divided by the carrying capacity of the culture (OD_600_). With all three QS reporters, a linear-fit model supports the data more than a step function fit (for *lasB*, AIC linear, 0; AIC step function, 281.55; for *pqsA*, AIC linear, 1.22; AIC step function, 283.43; and for *rhlI*, AIC linear, 40.68; AIC step function, 351.86). Download FIG S7, DOCX file, 0.1 MB. Copyright © 2022 Rattray et al. 2022 Rattray et al. https://creativecommons.org/licenses/by/4.0/ This content is distributed under the terms of the Creative Commons Attribution 4.0 International license.

## DISCUSSION

Our results show that populations of P. aeruginosa can respond in a smoothly graded manner to variations in environmental density (Fig. 2), that populations exhibit significant bimodality at higher densities (Fig. 3), and that this population scale-graded response can be described by the number of responsive ON cells and the intensity of the ON state (Fig. 4). The ability to achieve a graded population scale response implies in principle that P. aeruginosa can tune collective responses (such as the secreted elastase virulence factor produced by our *lasB* reporter) to graded environmental changes, rather than simply course graining into a simple “high/low” dichotomy. A similar population scale-graded response to continuous environmental variation is visible in the data from Allen et al., who looked at variation in the genotypic composition of mixed populations grown to the same total density (13). As the proportion of wild-type (PAO1 versus Δ*lasR* cheats) increased, the wild-type per-capita investment in cooperative LasB secretions also increased, providing a simple behavioral mechanism to protect cooperative investments from exploitation by cheats (13, 22).

The existence of graded population scale responses across two continuously various environmental inputs (density and genotypic composition) raises the question, why use a graded response? Is there an evolutionary rationale for a graded response, or is a graded increase simply the “best approximation” of a threshold response, given a simple system working under genetic constraints? Existing evolutionary theory suggests that graded investment reaction norms can be adaptive under a range of distinct scenarios (49, 50). In the specific context of quorum-sensing bacteria, evolutionary theory suggests that population scale responses to increasing density should depend critically on the shape of the cost and benefit functions of increasing cooperative investments. Specifically, a graded response is predicted to be the optimal strategy if the benefit function is decelerating and costs are linear with increasing investment (51).

To further consider the functional context of the graded reaction norms, we turn to the single-cell-scale data, which reveal how the graded population response is built from the contributions of individual cells. In agreement with previous work with multiple quorum-sensing organisms (11, 30–41), we found cell scale heterogeneity. In addition, our results illustrate how cellular heterogeneity changes with the environment, demonstrating the onset of ON/OFF bimodality at intermediate densities, with both the proportion of cells ON and the intensity of the cellular ON states increasing with increases in culture carrying capacity (Fig. 3 and 4).

The presence of a bimodal QS response is in contrast with the common view of QS as a mechanism of cell synchronization. Scholz and Greenberg support the synchronization premise by showing that positive autoregulation synchronizes expression of the signal synthase gene (*lasI*) compared to populations with the synthase gene locked on (29). Yet the study by Scholz and Greenberg, along with many more single-cell QS papers (11, 30–41), demonstrates substantial heterogeneity across individual wild-type cells, with expression levels varying over orders of magnitude. Here, we report that cellular heterogeneity transitions from uni- to bimodal at high densities (Fig. 3). The observation of bimodal cellular responses at high densities is consistent with previous studies that implicitly reveal bimodal cellular responses (31–33, 40, 41). For example, Darch et al. report distinct populations of QS-responsive and nonresponsive P. aeruginosa cells within single experimental runs (32).

In principle, this bimodality could be due to variation in the rate at which cells encounter signal (extrinsic heterogeneity). However, our culturing parameters (shaken liquid) were chosen to reduce spatial heterogeneities and are similar to the design of Scholz and Greenberg (29). Alternatively, this bimodality could be due to heterogeneity in individual-cell response thresholds to a homogenous signal environment (intrinsic heterogeneity). The existence of heterogeneous response thresholds is also consistent with experimental studies documenting that signal supplementation can induce QS responses in some, but not all, cells (35, 41). Previous research on bimodal gene expression points to a number of regulatory features that are at play in the effector *lasB*, specifically multiple transcription factor binding sites and positive-feedback loops (52).

On an evolutionary scale, cellular heterogeneity is an evolvable trait that can in theory buffer populations against fluctuating environments (bet hedging) and/or provide benefits of specialization (division of labor) (53, 54). Recently, the presence of heterogeneous QS response at the single-cell scale has been ascribed to a potential bet hedge against rapidly changing environments where QS could shift from a beneficial to a nonbeneficial behavior (38), suggesting that our OFF cells were poised to more quickly resume growth in the event of a rapid return to a growth-friendly environment.

We made a number of specific observational choices in order to conduct our experiment that could have shaped our results in ways that are not generalizable to other contexts. In the supplemental material, we detail a number of additional experiments (and alternate statistical analysis approaches [https://github.com/GaTechBrownLab/Rattray-2022]) that collectively illustrate the robustness of our findings. In brief, we found that our single-cell results were not sensitive to the time the population was sampled (Fig. S5), the presence of a potentially leaky *Plac*::*lasR* on the pMHLAS construct (Fig. S6), or the plasmid nature of the pMHLAS construct (55, Fig. S6). Additionally, we recognize that *lasB* is only one gene out of hundreds that are controlled by QS (3) and is often coregulated by other factors (56–58). We chose to initially focus on *lasB* because it is a traditionally studied QS-controlled trait (59–61), it is under multisignal control (12, 42) and has clinical significance as a virulence factor (62, 63). To begin to address the generality of our results across genes in P. aeruginosa, we show that two other QS-regulated genes with complex promoters, *pqsA* and *rhlI*, also support a graded population response (Fig. S7). It remains to be seen whether the graded responses we report here are consistent across all QS-controlled genes in P. aeruginosa and across QS systems in other species.

A recent transcriptomic analysis of clinical versus *in vitro* gene expression in P. aeruginosa called into question the clinical relevance of *in vitro* models of QS, reporting that QS activity (including *lasB* expression) was systematically higher in *in vitro* models (64). Our results provide a simple interpretation of this difference: *in vitro* models are typically conducted under higher experimental densities, resulting in higher levels of average QS gene expression (Fig. 2). Consistent with this graded response interpretation, Cornforth et al. (64) also reported higher levels of relative expression in *in vitro* biofilm models (close-packed cells, the highest local density achievable) than in *in vitro* planktonic models.

In summary, our results provide a finely resolved mapping of the QS reaction norm to environmental density in PAO1, on both the collective and single-cell scales. On the population scale, we saw a graded linear response across a range of cellular densities (1 × 10^8^ cells/mL to 2 × 10^9^ cells/mL) and significant individual-scale bimodality at higher densities. We further resolved this linear population response (Fig. 2) into a combination of the likelihood of being responsive and the intensity of response (Fig. 4). In an infection context, our results indicate that there is no hard threshold separating a subquorate “stealth” mode and a quorate “attack” mode (65). One implication is that attempts to control virulence and biofilm expression in medicine and industry via QS inhibition could have impacts across a wider spectrum of population densities. In this applied context, it is important to assess the generality of our results and ask, how do QS reaction norms vary across strains and species of QS bacteria? How do they vary across environments? More broadly, our work undermines the threshold concept of a “quorum,” instead placing QS bacteria in the graded world of reaction norms.

## MATERIALS AND METHODS

### Bacterial strains and growth conditions.

The two main bacterial strains used in this study were P. aeruginosa NPAO1 (Nottingham-PAO1) containing the *PlasB*::*gfp*(*ASV*) quorum sensing reporter pMHLAS (47) and a double signal synthase mutant incapable of producing QS signal molecules, P. aeruginosa NPAO1 Δ*lasI* Δ*rhlI* containing the same *PlasB*::*gfp*(*ASV*) quorum sensing reporter, pMHLAS. A complete table of strains used can be found in Table S1. Overnight cultures were grown in lysogeny broth (LB) supplemented with 50 μg/mL gentamicin to maintain the pMHLAS plasmid, with shaking at 37°C. Experiments were conducted in lightly buffered (50 mM 3-(N-morpholino)propanesulfonic acid [MOPS]) M9 minimal defined media composed of an autoclaved basal salts solution (Na_2_HPO_4_, 6.8 g L^−1^; KH_2_PO_4_, 3.0 g L^−1^; NaCl, 0.5 g L^−1^) and filter-sterilized 1 mM MgSO_4_, 100 μM CaCl_2_, and 1× Hutner’s trace elements with casein digest as the sole carbon source (Thermo Fisher Difco casein digest; catalog no. 211610).

10.1128/mbio.00745-22.9 TABLE S1 Strains used in this study. Download Table S1, PDF file, 0.03 MB. Copyright © 2022 Rattray et al. 2022 Rattray et al. https://creativecommons.org/licenses/by/4.0/ This content is distributed under the terms of the Creative Commons Attribution 4.0 International license.

### Controlling culture carrying capacity.

We manipulated density by controlling the limiting resource in the media, carbon, allowing us to tune the carrying capacity of each treatment (Fig. S1). To cover a variety of densities, we generated a carbon range between 0.05% and 0.25% via dilutions of a 0.5% carbon minimal medium stock for a total of 13 different carrying capacities with three replicates each. This produced a range of densities environments from 1.18 × 10^8^ cells/mL to 2.02 × 10^9^ cells/mL. Overnight cultures were grown in LB with gentamicin at 50 μg/mL and centrifuged at 8,500 × *g* for 2 min. The cells were then washed twice with carbonless minimal medium, and then each carbon treatment was adjusted to an OD_600_ of 0.05. Then, 200 μL of each sample was added to a 96-well microplate. Plates were incubated with shaking at 37°C in a Cytation/BioSpa plate reader, and growth curves were generated by absorbance (OD_600_) readings taken at 30-min intervals.

### Measuring population QS response.

To measure population response, we performed growth curve experiments as previously described using PAO1 *PlasB*::*gfp*(*ASV*), additionally taking fluorescence readings at 30-min intervals. Fluorescence was recorded when populations reached the end of their exponential growth phase, before they entered stationary phase. Background fluorescence of the reporter was determined with the QS signal-deficient mutant PAO1 Δ*lasI* Δ*rhlI PlasB*::*gfp*(*ASV*). The population microplate data (Fig. S2) and averaged microscope data (Fig. 2) agreed, so the latter are provided in the primary text.

### Measuring individual QS response.

To measure individual response, we performed growth curve experiments as described above but removed samples for microscopy once cells reached end exponential phase. Since we controlled carrying capacity with the amount of carbon, the exact time that cells reached the end of exponential growth differed across treatments by 2 to 3 h. To robustly sample cultures at this specific point, the slope of the two most recent time points on the growth curve was monitored and samples were taken as the slope approached 0. Replicate wells were kept growing to confirm that the treatment entered stationary phase right after the sampling time point. We also determined that our results are generalizable even when sampling at a predetermined hour across concentrations (Fig. S5). Samples were stained with propidium iodide to differentiate between live and dead cells, and a small aliquot (5 μL) was added to a 0.01% poly-l-lysine-coated slide to immobilize cells and immediately imaged to avoid changes in expression between sample acquisition and imaging in the dark on a Nikon Eclipse TI inverted microscope at a magnification of ×20. Live-cell fluorescence microscopy was used for this study, as fluorophores can be sensitive to fixation/permeabilization. These techniques can result in a decrease in fluorescence and therefore decrease in the observable dynamic range. Bright-field, green fluorescence (20% Lumencor light engine power, 200-ms exposure, and 64× *g*ain [sufficient for imaging of low-fluorescence cells without saturating pixel intensity]), and red fluorescence (20% Lumencor light engine power, 800-ms exposure, and 64× *g*ain) channels were captured. Between 5,000 and 15,000 individual cells were captured for each sample. Aliquots were diluted immediately before imaging with carbonless minimal medium when required to ensure an even distribution of cells.

### Single-cell image analysis.

A custom macro in ImageJ was written to analyze the image data, outlined in Fig. S8. The macro uses ImageJ’s “analyze particles” command to identify single cells on the bright-field image. This generated a region of interest (ROI) for each individual cell, and these ROIs were overlaid onto the corresponding fluorescent image. The red fluorescence channel was used to identify dead cells with compromised membranes, which were excluded from further analysis. The green fluorescence channel reflected the QS reporter, and pixel intensity was measured as a proxy for level of QS response. This tabulated live cell expression data was then analyzed using Stata statistical software release 17 from StataCorp LLC. In order to improve the fit of the mixed models, the lowest pixel intensity measurement in the highest-carbon PAO1 Δ*lasI* Δ*rhlI PlasB*::*gfp*(*ASV*) treatment was subtracted from all pixel intensities so that expression started at 0.

10.1128/mbio.00745-22.8 FIG S8 Visual summary of single-cell microscopy analysis pipeline. (A) Phase-contrast image of individual cells immobilized on 0.01% poly-l-lysine-coated glass slides. (B) ImageJ’s “background subtraction” command was used to increase contrast between the cells and the background. (C) A mask of the phase-contrast channel was created using ImageJ’s default auto threshold, a variation of the IsoData algorithm. (D) ImageJ’s “analyze particles” command was then used to identify the features (cells) from the image, this generated a set of regions of interest (ROIs), shown in yellow (superimposed). (E) Unaltered fluorescence channel image of the same cells. (F) ROIs generated from the phase-contrast image were superimposed onto the unedited fluorescence channel image, either red fluorescence for dead-cell identification via propidium iodide or green florescence for the QS reporter, and ImageJ’s “measure” command was used to find the average pixel intensity within each ROI. Any ROIs with red fluorescence were identified as compromised cells and removed from further analysis. Download FIG S8, DOCX file, 2.2 MB. Copyright © 2022 Rattray et al. 2022 Rattray et al. https://creativecommons.org/licenses/by/4.0/ This content is distributed under the terms of the Creative Commons Attribution 4.0 International license.

### Statistical-analysis summary.

The analysis was done using Stata statistical software release 17 from StataCorp LLC and the additional third-party resources (66–70). Each of the 39 populations was fit to a finite mixture model of two gamma distributions. The latent classes in the mixture model correspond to OFF and ON cells. Gamma distributions are preferred to normal distributions, as gene expression is strictly nonnegative and necessarily right skewed. The models provide maximum likelihood estimates of the proportion of cells in each latent class and the shape and scale parameters of the component gamma distributions. Mean expression level for each distribution is the product of shape and scale parameters. Information criteria for aggregate mean expression level were also calculated using Stata. The analysis supplement is hosted at https://github.com/GaTechBrownLab/Rattray-2022.
