# Supplementary material for: Bacterial Quorum Sensing Allows Graded and Bimodal Cellular Responses to Variations in Population Density
Source: mBio. 2022 May 18;13(3):e00745-22. doi: 10.1128/mbio.00745-22 (PMC9239169; doi:10.1128/mbio.00745-22)
Supplement: FIG S1 [file mbio.00745-22-s0001.docx]

**Figure S1.** **Growth curves for PAO1 pMHLAS across different carbon limiting environments.** Discrete environmental densities can be generated by varying carbon availability, therefore manipulating the carrying capacity of the culture.
