# Supplementary material for: Bacterial Quorum Sensing Allows Graded and Bimodal Cellular Responses to Variations in Population Density
Source: mBio. 2022 May 18;13(3):e00745-22. doi: 10.1128/mbio.00745-22 (PMC9239169; doi:10.1128/mbio.00745-22)
Supplement: FIG S2 [file mbio.00745-22-s0002.docx]

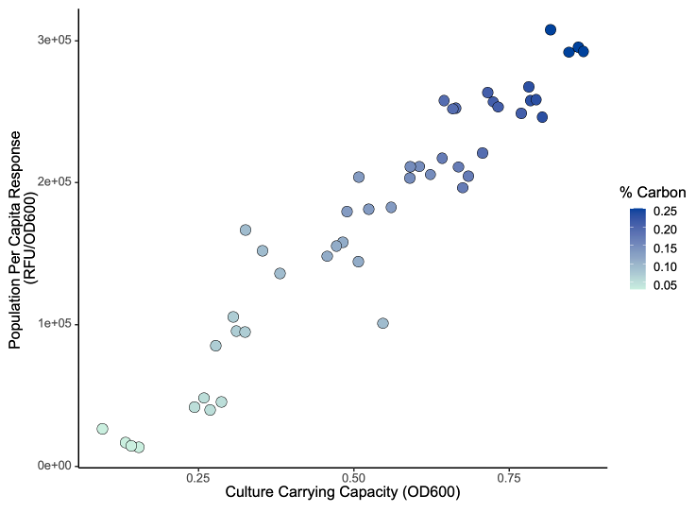


**Figure S2. Microplate population per capita data is graded and linear.** PAO1 pMHLAS was grown as mentioned in the methods and fluorescence and OD_600_ were measured on a Cytation Sense plate reader. Microplate results agree with microscopy results that that population response to increasing cell density is linear and graded. OD_600_ of 0.25 is 5.5x10^8^ cells/ml, OD_600_ of 0.50 is 1.3x10^9^ cells/ml, and OD_600_ of 0.75 is 2.05x10^9^ cells/ml.
