# Supplementary material for: Bacterial Quorum Sensing Allows Graded and Bimodal Cellular Responses to Variations in Population Density
Source: mBio. 2022 May 18;13(3):e00745-22. doi: 10.1128/mbio.00745-22 (PMC9239169; doi:10.1128/mbio.00745-22)
Supplement: FIG S3 [file mbio.00745-22-s0003.docx]

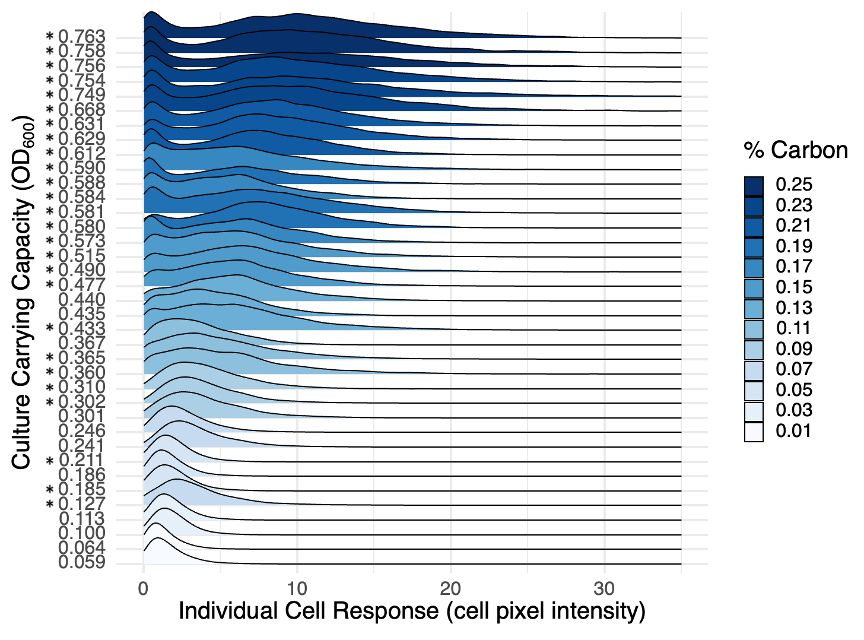


**Figure S3. Ridgeline density plot (bandwidth = 0.529) of single-cell *lasB* reporter response data** showing the distribution of individual cell QS expression across the population. All 39 replicates are plotted separately. Asterisks indicate significant bimodality (Hartingan’s Dip Test [49], Figure S4).
