# Supplementary material for: Bacterial Quorum Sensing Allows Graded and Bimodal Cellular Responses to Variations in Population Density
Source: mBio. 2022 May 18;13(3):e00745-22. doi: 10.1128/mbio.00745-22 (PMC9239169; doi:10.1128/mbio.00745-22)
Supplement: FIG S4 [file mbio.00745-22-s0004.docx]

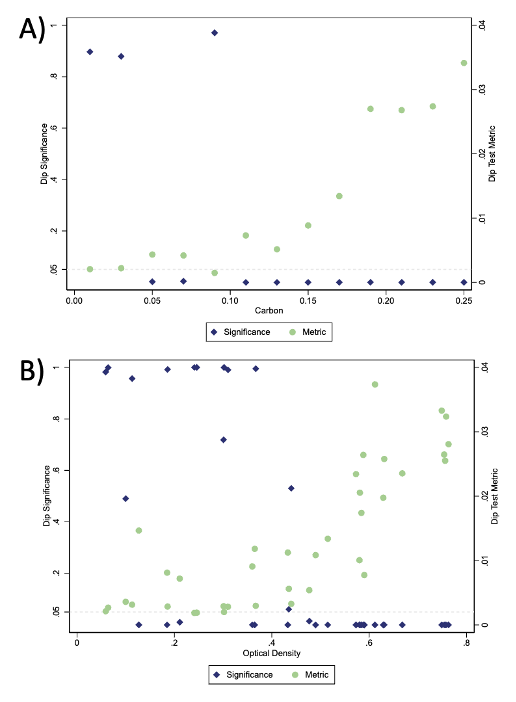


**Figure S4. Hartigan’s Dip Test.** A) Results of Hartigan's dip test [49] for assessing bimodality of the expression level distributions at each carbon level for data shown in Figure 4. B) Results of Hartigan's dip test for assessing bimodality of the expression level distributions at each OD level for data shown in Figure S3. Statistical significance in the form of p-value is plotted in yellow on the left axis, and the dip metric is plotted in green on the right axis. The most conservative p-value is shown.
