# Supplementary material for: Bacterial Quorum Sensing Allows Graded and Bimodal Cellular Responses to Variations in Population Density
Source: mBio. 2022 May 18;13(3):e00745-22. doi: 10.1128/mbio.00745-22 (PMC9239169; doi:10.1128/mbio.00745-22)
Supplement: FIG S5 [file mbio.00745-22-s0005.docx]

**
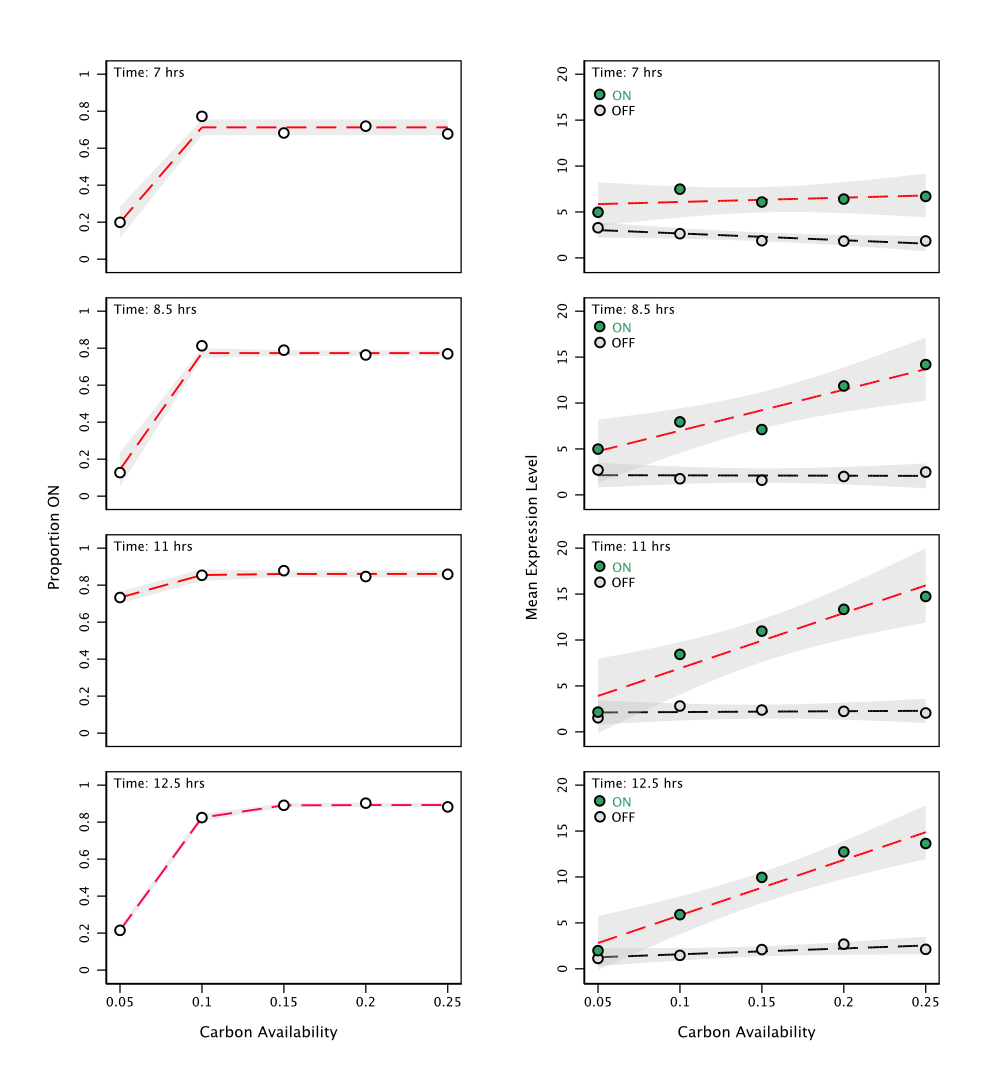
Figure S5. Single cell results are not sensitive to the exact time sampled.** In order to test the generality of our results and how sensitive they are to the specific time sampled, we repeated our main experiment with PAO1 pMHLAS, but took samples from five different time points instead of just entry into stationary phase. We observed the same bimodal response and shifts in proportion responding and level of response across all timepoints, concluding that our results are not sensitive to the exact measurement time.
