# Supplementary material for: Bacterial Quorum Sensing Allows Graded and Bimodal Cellular Responses to Variations in Population Density
Source: mBio. 2022 May 18;13(3):e00745-22. doi: 10.1128/mbio.00745-22 (PMC9239169; doi:10.1128/mbio.00745-22)
Supplement: FIG S6 [file mbio.00745-22-s0006.docx]

**
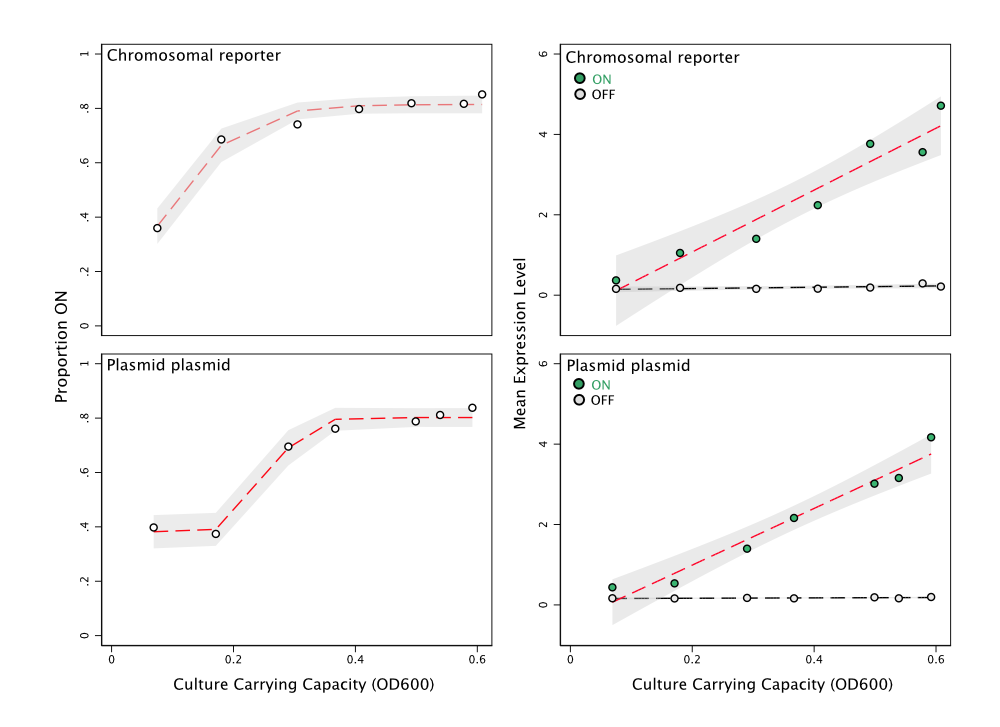
**

**Figure S6. Presence/absence of *Plac::lasR* and plasmid nature of the reporter do not impact graded QS response.** The plasmid hosting our reporter, pMHLAS, also contains P*lac::lasR.* While the lac operon is typically repressed, some strains of *Pseudomonas* are capable of leaky, non-induced expression [73]. To confirm our single cell data, we repeated the experiment using a strain with the P*lasB::gfp(ASV)* reporter inserted with the mini-Tn5 method. This strain does not contain P*lac::lasR* and expresses WT levels of *lasR*. We found the same bimodal response to density environments in both strains. This indicates that neither the presence of P*lac::lasR* nor the plasmid-based nature of our reporter are responsible for the graded QS response. This could mean either there is not an appreciable amount of *lasR* being made from the P*lac::lasR* or that the increase of *lasR* does not impact the observed response. These results confirm that the observed response across density is not sensitive to plasmid copy number or the presence of potentially leaky P*lac::lasR* with this specific reporter.
