# Supplementary material for: Bacterial Quorum Sensing Allows Graded and Bimodal Cellular Responses to Variations in Population Density
Source: mBio. 2022 May 18;13(3):e00745-22. doi: 10.1128/mbio.00745-22 (PMC9239169; doi:10.1128/mbio.00745-22)
Supplement: FIG S7 [file mbio.00745-22-s0007.docx]

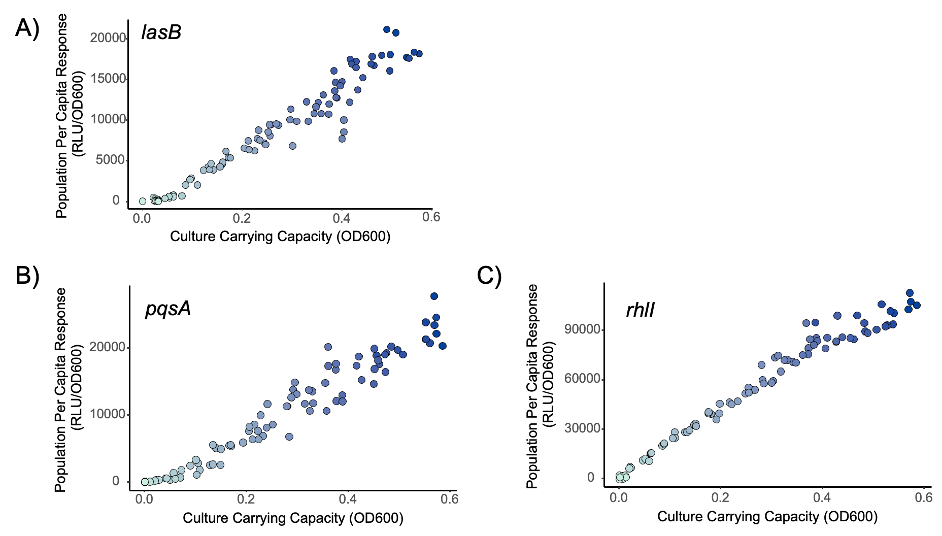


**Figure S7.** **Population response to increasing cell density using chromosomally inserted lux based QS reporters (72-74)**. A) strain P NPAO1 mini-CTX PlasB::lux, B) NPAO1 mini-CTX PpqsA::lux, and C) NPAO1 mini-CTX PrhlI::lux. Bacteria were grown following the same methods outlined in the main text methods and monitored in a plate reader. Per capita expression (RLU/OD_600_) was calculated by taking the maximum expression (RLU) divided by the carrying capacity of the culture (OD_600_). With all three QS reporters, a linear fit model supports the data more than a step-function fit (lasB, AIC linear: 0, AIC step-function: 281.55. pqsA, AIC linear: 1.22, AIC step-function: 283.43. rhlI, AIC linear: 40.68, AIC step-function: 351.86).
