# Supplementary material for: Bacterial Quorum Sensing Allows Graded and Bimodal Cellular Responses to Variations in Population Density
Source: mBio. 2022 May 18;13(3):e00745-22. doi: 10.1128/mbio.00745-22 (PMC9239169; doi:10.1128/mbio.00745-22)
Supplement: TABLE S1 [file mbio.00745-22-s0009.pdf]

**Strain/Plasmid**	**Relevant Characteristics**	**Figure**	**Source or Reference**
NPAO1 pMHLAS	QS reporter *PlasB::gfp(ASV), Plac::lasR*	2, 3, 4, S1, S2, S4, S5	Hentzer et al., 2002
NPAO1 *ΔlasIΔrhlI* pMHLAS	Double signal synthase mutant, QS reporter *PlasB::gfp(ASV)*	2, 3, 4	This paper
PAO1 MH340 mini-Tn5 *PlasB::gfp(ASV)*	QS reporter *PlasB::gfp(ASV)* inserted via the mini-Tn5 method	S5	Kasper Nørskov Kragh
NPAO1 mini-CTX P*lasB::lux*	luxCDABE based QS reporter inserted at the *attB* site	S6	This paper
NPAO1 mini-CTX P*pqsA::lux*	luxCDABE based QS reporter inserted at the *attB* site	S6	This paper
NPAO1 mini-CTX P*rhlI::lux*	luxCDABE based QS reporter inserted at the *attB* site	S6	This paper

Table S1. Strains used in this manuscript.
