# Supplementary material for: Bacterial Quorum Sensing Allows Graded and Bimodal Cellular Responses to Variations in Population Density
Source: mBio. 2022 May 18;13(3):e00745-22. doi: 10.1128/mbio.00745-22 (PMC9239169; doi:10.1128/mbio.00745-22)
Supplement: FIG S8 [file mbio.00745-22-s0008.docx]

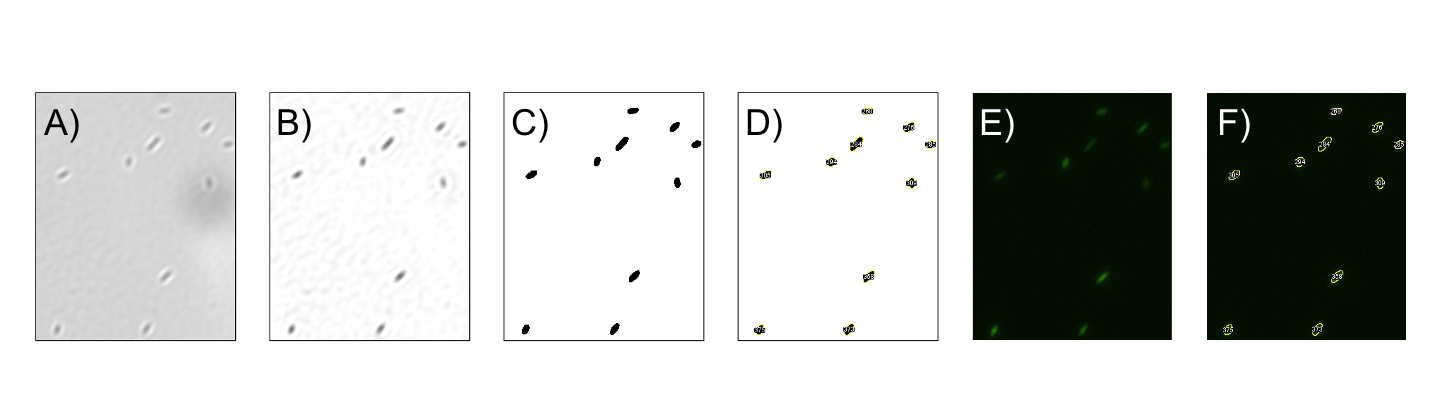


**Figure S8. Visual summary of single-cell microscopy analysis pipeline.** A) Phase contrast image of individual cells immobilized on 0.01% poly-l-lysine coated glass slides. B) ImageJ’s “background subtraction” command was used to increase contrast between the cells and the background. C) A mask of the phase contrast channel was then created using ImageJ’s default auto threshold, a variation of the IsoData algorithm. D) ImageJ’s “analyze particles” command was then used to identify the features (cells) from the image, this generates a set of regions of interest (ROIs), shown in yellow superimposed. E) Unaltered fluorescence channel image of the same cells. F) ROIs generated from the phase contrast image are then be superimposed onto the unedited fluorescence channel image, either red fluorescence for dead cell identification via propidium iodide or green florescence for the QS reporter, and ImageJ’s “measure” command was used to find the average pixel intensity within each ROI. Any ROIs with red fluorescence were identified as compromised cells and removed from further analysis.
